# *AK8* deficiency causes primary ciliary dyskinesia by disrupting energy homeostasis in the airways

**DOI:** 10.1093/ajrccm/aamag214

**Published:** 2026-04-28

**Authors:** Dörthe Thüß, Ying Liu, Gerard W Dougherty, Ellie J Maas, Jie He, Xianglin Zhou, André Schultz, Kim G Nielsen, Heymut Omran, Hong Luo, the AK8 Study Group, Aideen M McInerney-Leo, Binyi Yang, Danhui Yang, Cheng Lei, Ting Guo, Manqing Guo, Hui Fan, Shuizi Ding, Lv Liu, Emma L Duncan, Kai Wohlgemuth, Diana C Bracht, Heike Olbrich, Niki T Loges, Cynthia Rieck, Alina Wilken, Julia König, Rim Hjeij, Bernd Dworniczak, Johanna Raidt, Morten Dunø, Mathias G Holgersen, June K Marthin, Anne B Chang, Isabell Ricklefs, Alexander Herz, Wiebke Hagedorn, Folke Brinkman, Dominik Otto

**Affiliations:** Department of General Pediatrics, University Hospital Muenster, Muenster, Germany; Department of Pulmonary and Critical Care Medicine, Second Xiangya Hospital, Central South University, Changsha, Hunan, China; Changsha Human Ciliary Diseases and Translational Medicine Innovation Center, Second Xiangya Hospital, Central South University, Changsha, Hunan, China; Department of General Pediatrics, University Hospital Muenster, Muenster, Germany; Dermatology Research Centre, Frazer Institute, University of Queensland, Brisbane, Queensland, Australia; Department of Pulmonary and Critical Care Medicine, Second Xiangya Hospital, Central South University, Changsha, Hunan, China; Changsha Human Ciliary Diseases and Translational Medicine Innovation Center, Second Xiangya Hospital, Central South University, Changsha, Hunan, China; Department of Pulmonary and Critical Care Medicine, Second Xiangya Hospital, Central South University, Changsha, Hunan, China; Changsha Human Ciliary Diseases and Translational Medicine Innovation Center, Second Xiangya Hospital, Central South University, Changsha, Hunan, China; Wal-yan Respiratory Research Centre, Kids Research Institute Australia, University of Western Australia, Perth, Western Australia, Australia; Department of Respiratory Medicine, Perth Children’s Hospital, Perth, Western Australia, Australia; Department of Pediatrics and Adolescent Medicine, Danish PCD Centre, Danish Pediatric Pulmonary Service, Rigshospitalet, Copenhagen University Hospital, Copenhagen, Denmark; Institute of Clinical Medicine, University of Copenhagen Faculty of Health and Medical Sciences, Copenhagen, Denmark; Department of General Pediatrics, University Hospital Muenster, Muenster, Germany; Department of Pulmonary and Critical Care Medicine, Second Xiangya Hospital, Central South University, Changsha, Hunan, China; Changsha Human Ciliary Diseases and Translational Medicine Innovation Center, Second Xiangya Hospital, Central South University, Changsha, Hunan, China

To the Editor:

Primary ciliary dyskinesia (PCD; MIM#244400) is a rare, chronic respiratory disorder that impairs motile ciliary beating, typically manifesting as recurrent respiratory tract infections, chronic rhinosinusitis (CRS), otitis media (OME), bronchiectasis, and variable lung function decline as well as infertility.^1^^,^^2^ Per recent guidelines from the American Thoracic Society and European Respiratory Society, establishing a PCD diagnosis requires a multitest pathway: transmission electron microscopy (TEM) and/or genetic testing can confirm the diagnosis but may require adjunct tests such as nasal nitric oxide (nNO) measurement, high-speed videomicroscopy, and immunofluorescence microscopy (IF).^3^ Approximately 30% of PCD cases lack a genetically confirmed diagnosis, underscoring the need for genetic discovery.^1^^,^^2^ Suspected PCD cases lacking both ciliary ultrastructural defects by TEM and a genetic diagnosis present a particular challenge. Furthermore, the genetic diagnosis may be confounded by variants of uncertain significance (VUS) that require validation.

Here, we sought to identify novel genetic defects in suspected PCD individuals excluded for variants in known PCD-causing genes. Inclusion criteria were chronic respiratory symptoms including CRS, OME, and recurrent lung infections. Individuals with or without bronchiectasis, situs abnormalities (eg, *situs inversus totalis*), and low nNO production rates (<77 nanoliters per minute) were included (*n* = 1301 exomes analyzed). Written informed consent was obtained from study individuals and family members according to protocols approved by the ethics committees of collaborating institutions, and the study complied with all ethical regulations from respective institutions.

We identified 3 individuals with homozygous, loss-of-function variants in adenylate kinase 8 (*AK8*) by whole-exome sequencing (WES) (Table 1 and Figure 1). Adenylate kinases, which maintain energy homeostasis by replenishing adenosine triphosphate, are long implicated in serving the high energy demands of ciliary beating.^4^^,^^5^ A recent study described an infertile male with asthenozoospermia due to pathogenic *AK8* variants but did not address any respiratory phenotype.^6^ To our knowledge, *AK8* deficiency has not been associated with chronic respiratory symptoms or a PCD diagnosis in humans.

**Figure 1:**
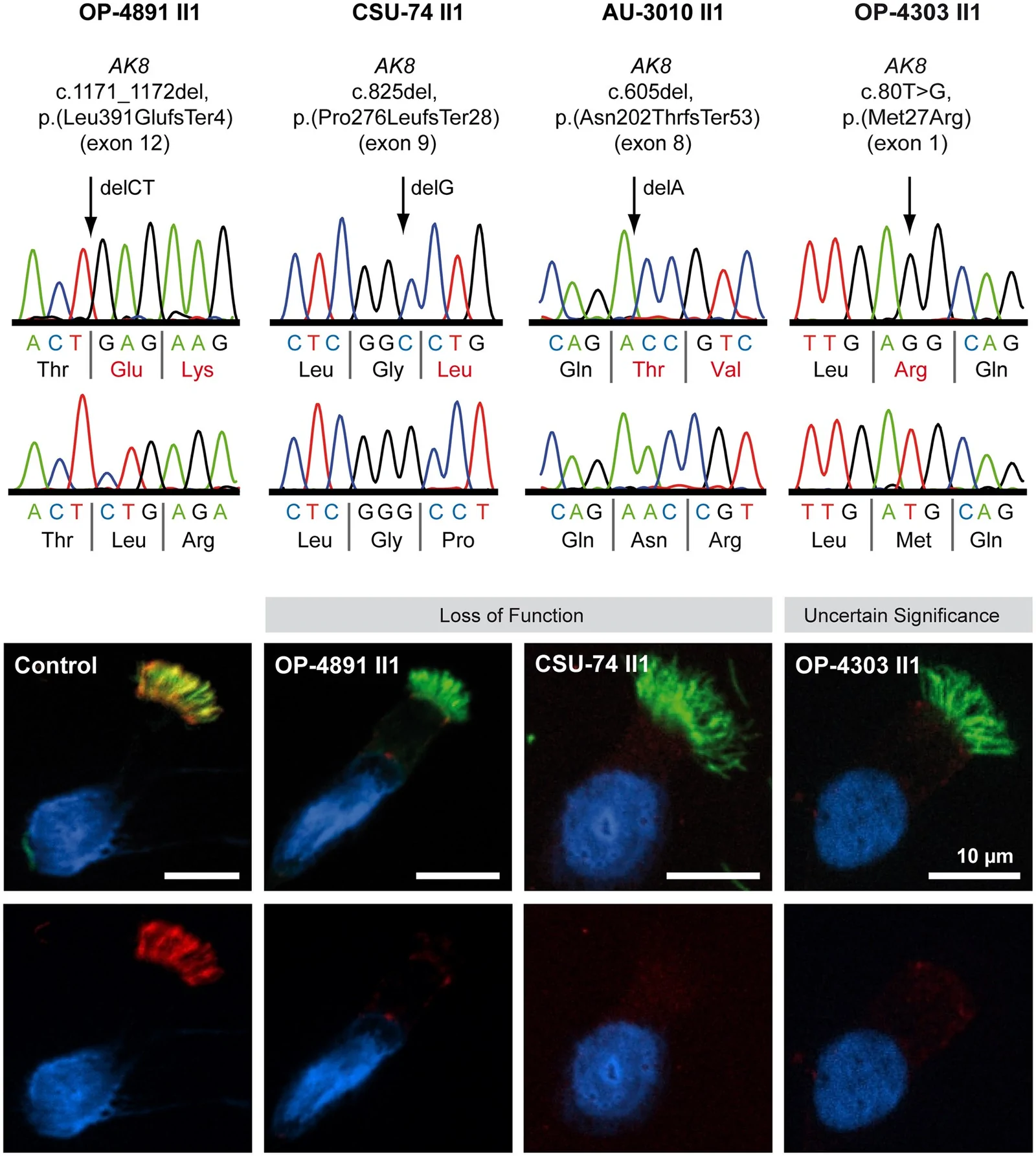
Genetic and immunofluorescence microscopy (IF) analysis confirm disease-causing *AK8* variants in suspected primary ciliary dyskinesia (PCD) individuals. Sanger sequencing confirms ultrarare, homozygous loss-of-function adenylate kinase 8 (*AK8*) variants in OP-4891 II1, CSU-74 II1, and AU-3010 II1 as well as a homozygous variant of uncertain significance (VUS) in OP-4303 II1. This ultrarare VUS has conflicting in silico pathogenicity predictions (https://varsome.com). Top chromatograms show DNA sequence of PCD individuals; bottom chromatograms show DNA sequence of healthy control individuals. RefSeq for *AK8* is NM_152572.3. Targeted IF confirms loss of AK8 protein in respiratory cilia from individuals with *AK8* variants predicting loss-of-function and uncertain significance. Healthy controls demonstrate panaxonemal localization of AK8 (red) throughout respiratory cilia (green) marked by acetylated tubulin. In contrast, AK8 (red) is not detectable in respiratory cilia from individuals OP-4891 II1 and CSU-74 II1 (who harbor loss-of-function *AK8* variants) as well as individual OP-4303 II1, who harbors an ultrarare, homozygous VUS in *AK8*. Images are merged with Hoechst 33342 staining to indicate nuclei with (top panel) or without (bottom panel) anti-acetylated tubulin (green). Immunofluorescence microscopy staining with polyclonal anti-AK8 antibody is performed as described.^7^ Individual AU-3010 II1 is not analyzed by IF.

**Table 1:** Overview of PCD individuals in this study.

Individual	OP-4891 II1	CSU-74 II1	AU-3010 II1	OP-4303 II1
**Age, y**	49	36	18	16
***AK8* variant**	c.1171_1172del, p.(Leu391GlufsTer4)	c.825del, p.(Pro276LeufsTer28)	c.605del, p.(Asn202ThrfsTer53)	c.80T > G, p.(Met27Arg)
**Minor allele frequency**	N/A	0.000004342	0.0002732	N/A
**SNP**	N/A	rs781774397	rs772875208	N/A
**Genetic method**	WES	WES	WES	Panel
**RDS**	No	No	Yes	No
**nNO**	34.3	12.6	75.6	54.3
**CRS**	Yes	Yes	Yes	Yes
**OME**	Yes	Yes	Yes	Yes
**Wet cough**	Yes	Yes	Yes	Yes
**Bronchitis**	Yes	Yes	Yes	Yes
**Bronchiectasis**	Yes	Yes	No	No
**FEV_1_**	105%	76%	98%	83%
**FVC**	96%	95%	106%	85%
**TEM**	Normal	Normal	Normal	Normal
**HSVM**	Abnormal CBP	Abnormal CBP	N/A	N/A
**Diagnostic IF**	Normal	Normal	N/A	Normal
**Targeted IF**	AK8 absent	AK8 absent	N/A	AK8 absent

Abbreviations: AK8, adenylate kinase 8; CBF, ciliary beat frequency; CBP, ciliary beat pattern; CRS, chronic rhinosinusitis; HSVM, high-speed videomicroscopy; IF, immunofluorescence microscopy; max, maximum; min, minimum; N/A, data not available; OME, chronic otitis media; PCD, primary ciliary dyskinesia; RDS, neonatal respiratory distress syndrome; TEM, transmission electron microscopy; WES, whole-exome sequencing.

a All individuals (current age in years) harbor ultrarare, homozygous *AK8* variants confirmed by Sanger sequencing. All individuals report chronic respiratory symptoms and recurrent lung infections. Bronchiectasis is present in OP-4891 II1 and CSU-74 II1; AU-3010 II1 and OP-4303 II1 show airway wall thickening but not bronchiectasis. Nasal nitric oxide production rates are within the diagnostic range to consider PCD (<77 nanoliters per minute). Forced expiratory volume in 1 second (FEV_1_) and FVC are specified in percent. Transmission electron microscopy shows normal ciliary ultrastructure (OP-4891 II1, 37 cross sections; CSU-74 II1, 50 cross sections; OP-4303 II1, 6 cross sections). High-speed videomicroscopy shows reduced CBFs within normal range: OP-4891 II1 (min-max = 4.5-5.5 Hz, at 25°C) vs healthy control pool (median = 6.36 Hz, min-max = 4.25-11.63 Hz, at 25°C); CSU-74 II1 (median = 10.3 Hz, min-max = 8.8-13.1 Hz, at 37°C) vs healthy control pool (median = 12.7 Hz, min-max = 9.3-13.7 Hz, at 37°C) with rotatory CBP in some fields for OP-4891 II1 and CSU-74 II1. Normal diagnostic IF indicates preserved axonemal localization of DNAH5, GAS8, and RSPH9. OP-4891 II1 reported infertility but fathered healthy twin daughters by in vitro fertilization. CSU-74 II1 reported oligo-asthenozoospermia but conceived a child (confirmed by genetic testing) without assisted reproduction technology.

Individual OP-4891 II1, a 49-year-old male, presented with CRS, OME, and pronounced mouth breathing refractory to nasal steroid treatment as well as chronic wet cough since childhood. Individual CSU-74 II1, a 36-year-old male, presented with CRS, OME, chronic wet cough, and purulent sputum. Individual AU-3010 II1, an 18-year-old male, presented with CRS and suppurative OME and reported nasal congestion and chronic wet cough since infancy. Computed tomography scans of the chest revealed bronchiectasis in the central and basal lung segments with airway wall thickening in OP-4891 II1 and CSU-74 II1, whereas diffuse small airway thickening without bronchial dilatation was observed in AU-3010 II1. Computed tomography scans of the sinus demonstrated mucosal thickening of the maxillary sinuses in all 3 individuals. Based on these findings, we incorporated *AK8* into our diagnostic PCD gene panel, which is more cost-effective and provides greater sequencing depth than standard WES, and subsequently identified a homozygous *AK8* missense variant in individual OP-4303 II1, a 16-year-old-male who previously tested negative by PCD panel. He reported CRS, OME, and chronic wet cough. Notably, all 4 male individuals have normal positioning of the thoracic and abdominal organs (*situs solitus*).

We previously showed that AK8 is robustly detectable in human respiratory cilia by IF and therefore examined AK8 in respiratory cilia of these individuals as described.^7^ Targeted IF revealed that AK8 was undetectable in respiratory cilia of individuals OP-4891 II1 and CSU-74 II1 in contrast to healthy control (Figure 1), thus confirming the functional impact of these variants predicting premature termination of translation. We also analyzed respiratory cilia from individual OP-4303 II1, who harbors homozygous *AK8* variant c.80T > G, p.(Met27Arg), which is classified as VUS. Notably, AK8 was not detectable in respiratory cilia from OP-4303 II1 (Figure 1), thus providing strong evidence at the protein level that this variant is disease-causing.

Diagnostic IF for representative axonemal structural proteins including GAS8 (nexin-link/dynein regulatory complex), DNAH5 (outer dynein arm), and RSPH9 (radial spoke or RS complex) showed normal ciliary localization in respiratory cilia from individuals OP-4891 II1, CSU-74 II1, and OP-4303 II1 comparable to healthy controls (Table 1). Consistent with these findings, TEM revealed normal respiratory ciliary ultrastructure in these individuals (Table 1). These data suggested that AK8 deficiency does not alter the major structural components of the axoneme at the molecular or ultrastructural level. High-speed videomicroscopy revealed that the ciliary beat frequency in individuals OP-4891 II1 and CSU-74 II1 was within the lower normal range of pooled healthy controls (Table 1). However, unlike the coordinated ciliary beating observed in healthy controls, cilia from individuals CSU-74 II1 and OP-4891 II1 exhibited some fields with an abnormal, rotatory ciliary beat pattern (CBP) discernible by top view.

Although *Ak8*^-/-^ mice have been reported previously,^8^ existing models differ in genetic strategy and reported phenotypes. We generated novel *Ak8*^-/-^ mice by deleting exons 7-11 using CRISPR-Cas9. Our *Ak8*^-/-^ model exhibited features similar to AK8-deficient PCD individuals, including CRS (mucus accumulation in the maxillary sinus), respiratory cilia with a rotatory CBP despite normal ultrastructure, and male infertility. Consistent with other *Ak8*^-/-^ models and the PCD individuals in this study, our mice did not display any features of *situs inversus* or heterotaxy.

A rotatory CBP is characteristic of individuals with genetic defects in the RS complexes.^9^ Recent studies including those by Bicka et al. have placed AK8 at the RS structure.^8^^,^^10^ Our IF analyses did not reveal loss of other canonical RS components (RSPH1, RSPH3, RSPH4A, and DNAJB13) in *AK8*-deficient cilia. This suggests that *AK8* deficiency does not abolish RS assembly but may impair RS function by rendering nearby complexes metabolically ineffective, producing an abnormal CBP that in turn impairs mucociliary clearance.

Overall, this report underscores the necessity of genetic discovery and testing to establish a PCD diagnosis in cases lacking distinguishing features such as *situs inversus* and ultrastructural defects by TEM. This also highlights the utility of adjunct targeted IF to validate the genetic diagnosis, identifies an essential role for AK8 in respiratory cell function and mucociliary clearance, and marks the first example of an adenylate kinase deficiency underlying chronic respiratory disease.

## Supplementary Material

aamag214_Supplementary_Data
